# *Endothiodon* cf. *bathystoma* (Synapsida: Dicynodontia) bony labyrinth anatomy, variation and body mass estimates

**DOI:** 10.1371/journal.pone.0189883

**Published:** 2018-03-14

**Authors:** Ricardo Araújo, Vincent Fernandez, Richard D. Rabbitt, Eric G. Ekdale, Miguel T. Antunes, Rui Castanhinha, Jörg Fröbisch, Rui M. S. Martins

**Affiliations:** 1 Instituto de Plasmas e Fusão Nuclear, Instituto Superior Técnico, Universidade de Lisboa, Lisboa, Portugal; 2 Museum für Naturkunde - Leibniz-Institut für Evolutions- und Biodiversitätsforschung, Berlin, Germany; 3 Huffington Department of Earth Sciences, SMU, Dallas, Texas, United States of America; 4 GEAL - Museu da Lourinhã, Lourinhã, Portugal; 5 Laboratoire de Paléontologie, Institut des Sciences de l’Évolution de Montpellier (ISE-M, UMR 5554, CNRS/UM/IRD/EPHE), Université de Montpellier, Montpellier, France; 6 European Synchrotron Radiation Facility, Grenoble, France; 7 Department of Bioengineering, University of Utah, Salt Lake City, United States of America; 8 Department of Biology, San Diego State University/San Diego Natural History Museum, San Diego, United States of America; 9 Academia das Ciências de Lisboa, Lisboa, Portugal; 10 GeoBioTec, Faculdade de Ciências e Tecnologia, Universidade Nova de Lisboa, Caparica, Portugal; 11 Instituto Gulbenkian de Ciência, Oeiras, Portugal; 12 LATR/IST/CTN - Campus Tecnológico e Nuclear, Bobadela, Portugal; 13 Institut für Biologie, Humboldt-Universität zu Berlin, Berlin, Germany; 14 CENIMAT/I3N, Faculdade de Ciências e Tecnologia, Universidade Nova de Lisboa, Caparica, Portugal; Monash University, AUSTRALIA

## Abstract

The semicircular canal (SC) system of the inner ear detects head angular accelerations and is essential for navigation and spatial awareness in vertebrates. Because the bony labyrinth encloses the membranous labyrinth SCs, it can be used as a proxy for animal behavior. The bony labyrinth of dicynodonts, a clade of herbivorous non-mammalian synapsids, has only been described in a handful of individuals and remains particularly obscure. Here we describe the bony labyrinth anatomy of three *Endothiodon* cf. *bathystoma* specimens from Mozambique based on digital reconstructions from propagation phase-contrast synchrotron micro-computed tomography. We compare these findings with the bony labyrinth anatomy of their close relative *Niassodon*. The bony labyrinths of *Endothiodon* and *Niassodon* are relatively similar and show only differences in the shape of the horizontal SCs and the orientation of the vertical SCs. When compared to extant mammals, *Endothiodon* and *Niassodon* have highly eccentric SCs. In addition, the *Endothiodon* SCs are nearly orthogonal. An eccentric and orthogonal SC morphology is consistent with a specialization in rapid head movements, which are typical of foraging or feeding behaviors. Furthermore, we estimate the body mass of these *Endothiodon* specimens at ~116 to 182 kg, based on the average SC radii calculated using a linear regression model optimized by the Amemiya Prediction Criterion. Our findings provide novel insights into the paleobiology of *Endothiodon* which are consistent with the peculiar feeding mechanism among dicynodonts presumed from their multiple postcanine toothrows.

## Introduction

The semicircular canal (SC) system of the inner ear has a fundamental role in proprioception by encoding rotations of the head [1–5]. The development of computed tomography has significantly increased our knowledge of the anatomy of the bony labyrinth in extinct and extant taxa, from reptilians to synapsids. It is currently accepted that the three-dimensional morphology of the bony labyrinth provides an ecomorphological signal, because it reflects how sensitive the inner ear is to angular motion of the head [6–11]. Thus, the analysis of fossilized inner ears can provide important insights into the lifestyle of extinct species (e.g., [12–14]). Furthermore, labyrinth anatomy and morphometrics are also useful for systematic purposes [15, 16].

Although a vast amount of information on mammaliaform labyrinth morphological diversity has accumulated over recent years (e.g., [17–23]), few studies have addressed the vestibular anatomy of non-mammaliaform synapsids (e.g., [24–27]). For instance, dicynodont osseous labyrinths have only been described in a small number of specimens [27–38] representing a minor subset of the enormous diversity of more than 120 dicynodont species currently known [39]. Furthermore, as most of these anatomical descriptions relied on imprecise reconstructions from serial grinding, rigorous morphological comparisons of non-mammaliaform synapsid bony labyrinths have remained challenging. This technical limitation has also hindered comparisons with extant species to address ecomorphological questions, for instance. Contrary to the view that the inner ear has conserved morphology, significant variations have been reported in some extant species, even at the intraspecific level (e.g., [40, 41]). This morphological diversity provides information about the animals’ lifestyles.

In this study, we used synchrotron radiation-based micro-computed tomography to examine the anatomy of the bony labyrinth of three rare specimens of the extinct Mozambican *Endothiodon* cf. *bathystoma* [42] and *Niassodon* [35] collected from the K5 Formation [43, 44] of the Metangula Graben, Niassa Province, Mozambique. Our results revealed unusually high SC eccentricity in *Endothiodon* and *Niassodon*, when compared to extant animals of the synapsid lineage. Interestingly, the unusual SC morphology of these Endothiodontia allowed us to explore a previously poorly known region of the bony labyrinth morphospace [45]. Furthermore, we found low intraspecific variation in the SC system among these *Endothiodon* specimens. Finally, we used the dimensions of the SC radii to estimate the body mass for *Endothiodon* with a highly-significant linear regression model.

## Materials and methods

### Institutional acronyms

MTA/ACL, Academia das Ciências de Lisboa, Lisbon, Portugal; AMNH, American Museum of Natural History, New York, United States of America; GPIT/RE, Institut und Museum für Geologie und Paläontologie, Tübingen, Germany; MB.R, Museum für Naturkunde, Leibniz-Institut für Evolutions- und Biodiversitätsforschung, Berlin, Germany; SAM-PK, Iziko South African Museum, Cape Town, Republic of South Africa.

### Materials

**Fig 1:**
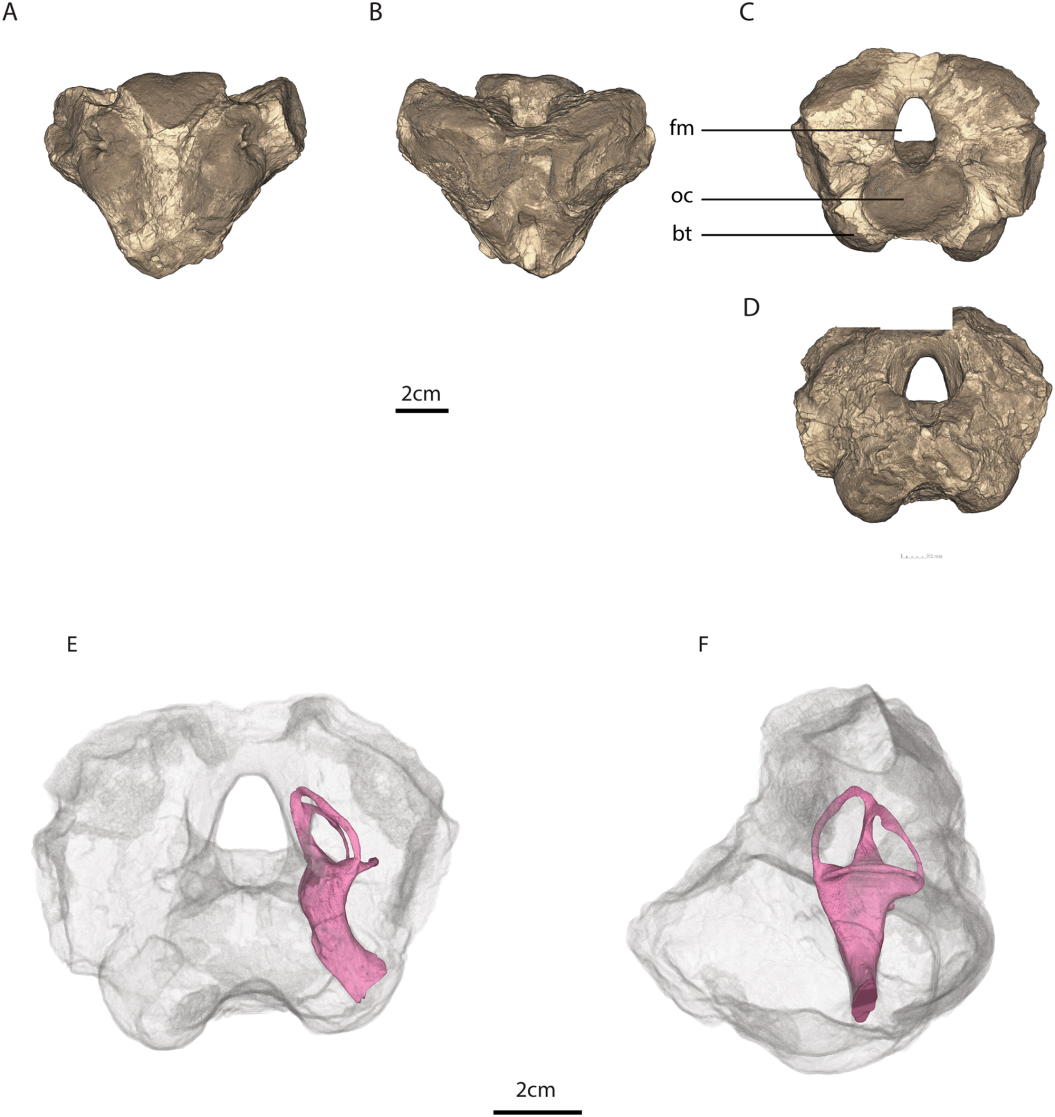
MTA/ACL001 basicranium. A, ventral, B, dorsal, C, posterior, and E, anterior views. Osseous labyrinth within the basicranium in E, anterior and F, left lateral views. Legend: bt, basal tubera, fm, foramen magnum, oc, occipital condyle.

**Fig 2:**
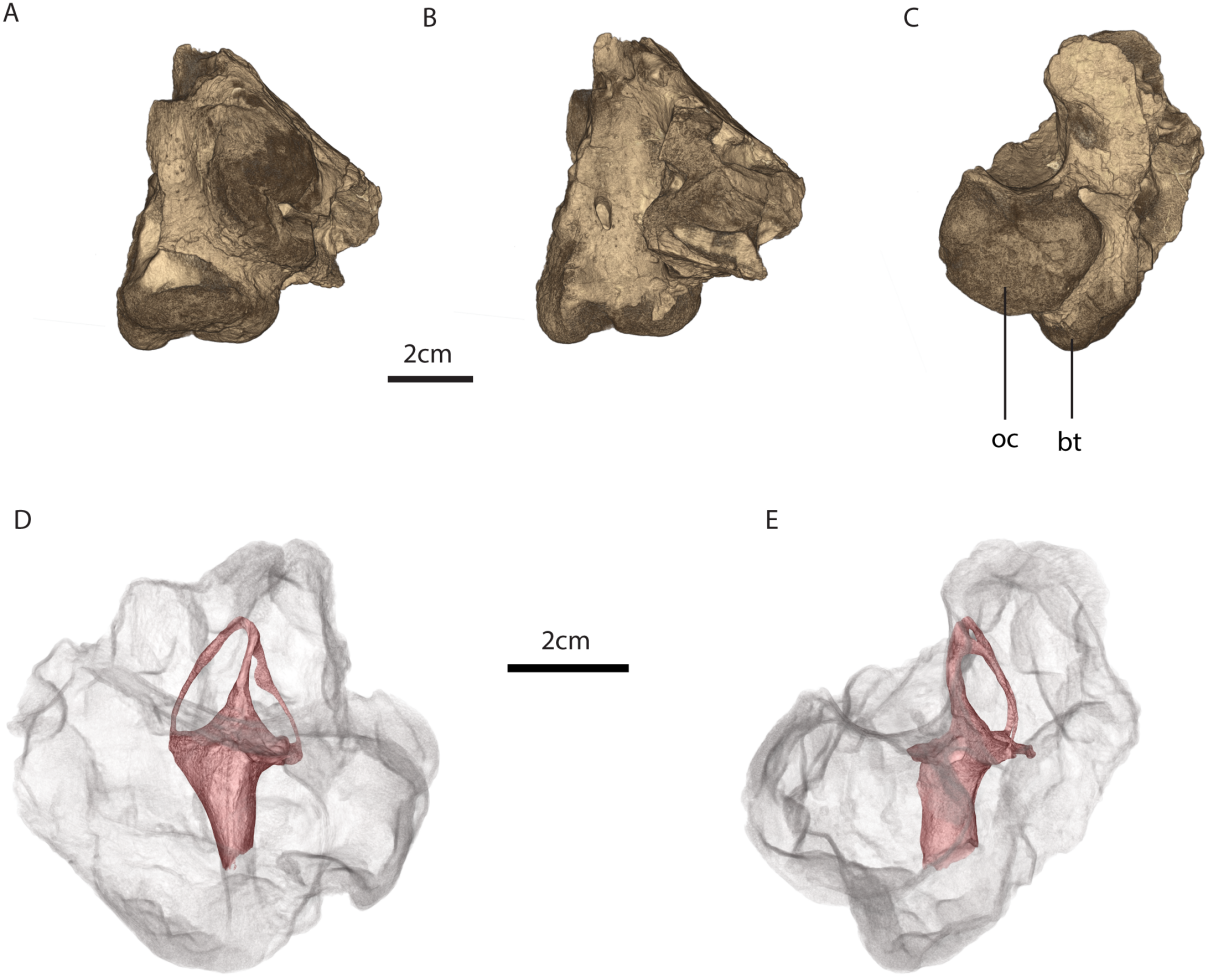
MTA/ACL002 basicranium. A, ventral view, B, dorsal view, C, posterior view, D, medial view with the osseous labyrinth, E, posterior view with the osseous labyrinth. Legend: bt, basal tubera, oc, occipital condyle.

**Fig 3:**
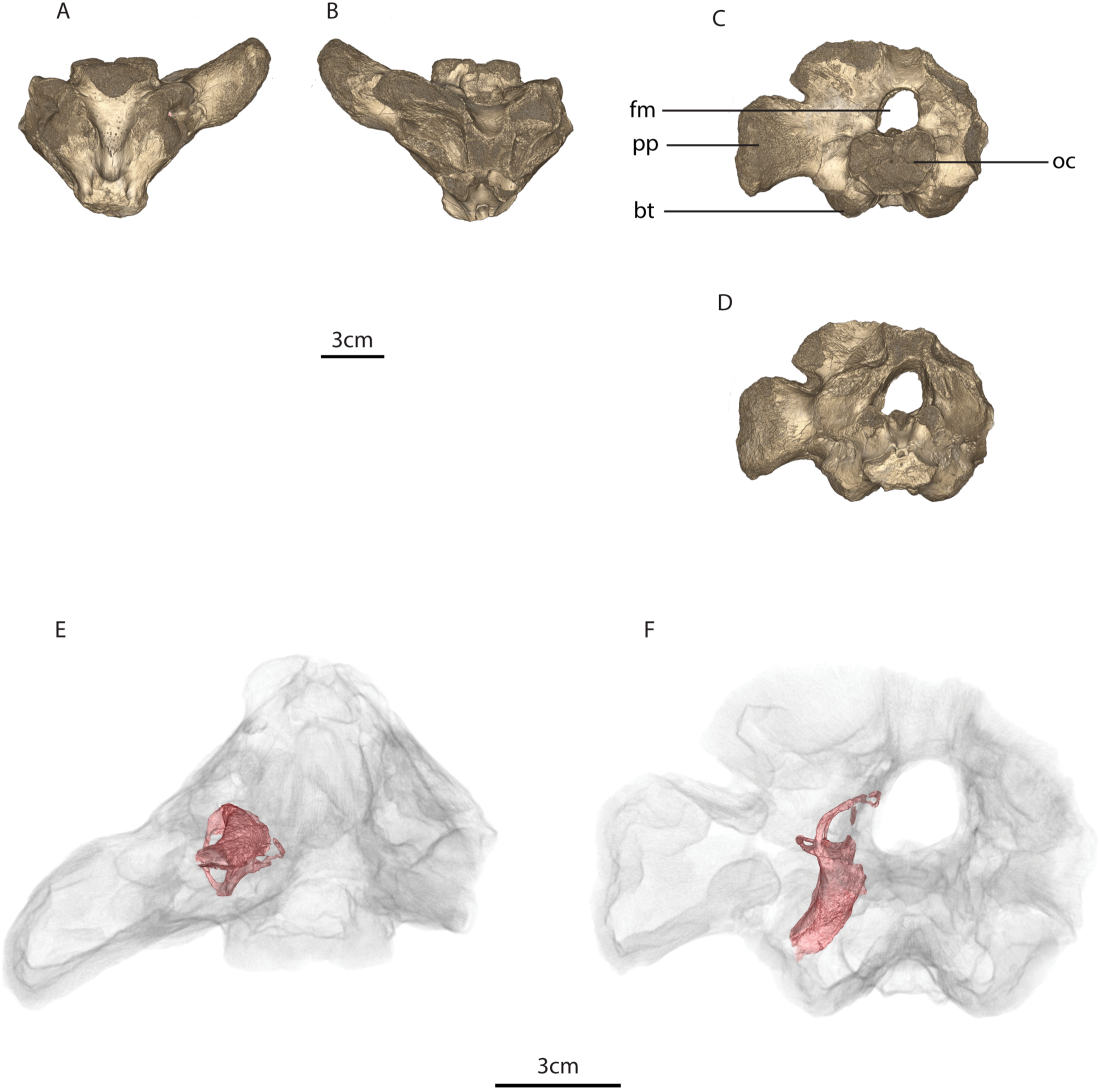
MTA/ACL003 basicranium. A, ventral view, B, dorsal view, C, posterior view, D, anterior view. Osseous labyrinth within the basicranium in E, ventral view, F, anterior view. Legend: bt, basal tubera, fm, foramen magnum, oc, occipital condyle, pp, paroccipital process.

We examined three rare and fragile *Endothiodon* cf. *bathystoma* specimens that preserve partial occipital and basicranial regions, namely MTA/ACL001 (Fig 1), MTA/ACL002 (Fig 2), MTA/ACL003 (Fig 3) [42]. The *Endothiodon* specimens can be consulted at the Academia de Ciências de Lisboa. The specimens were all collected from the K5 Formation [43, 44] of the Metangula Graben, Niassa Province, Mozambique. *Niassodon* is permanently deposited in the Museu Nacional de Geologia (Mozambique) collections. A comprehensive review of the history of the specimens and the collection of fossil vertebrates from Mozambique is described in the Supporting Information.

MTA/ACL001 is composed of the ventral portion of the supraoccipital enclosing the foramen magnum, the basioccipital fused to the exoccipitals with the occipital condyle eroded, the posterior portion of the basisphenoid with relatively intact basisphenoid tubera, and only the medial portions of the opisthotic (Fig 1).

MTA/ACL002 contains of a small fragment of the right portion of the supraoccipital and the most medial portion of the right opisthotic perforated by the right jugular foramen. MTA/ACL002 also contains the basioccipital, which forms together with the exoccipitals a tripartite occipital condyle, the latter being pierced by two roots of the hypoglossal foramina (Fig 2).

MTA/ACL003 is composed of the right opisthotic, the ventral portion of the supraoccipital, and the basioccipital, although the occipital condyle is eroded. In MTA/ACL003 the exoccipitals are fused to the basioccipital and there is a portion of the basisphenoid whose basisphenoidal tubera was largely eroded. In all specimens due to modern-day erosion an outer layer of the bone surface was significantly demineralized.

AMNH6156 natural cast of the inner ear was found to possess similar morphology to the *Endothiodon* inner ear endocasts, therefore, we incorporated a description and comparisons in S1 Text.

### Propagation phase-contrast synchrotron micro-computed tomography and segmentation

**Fig 4:**
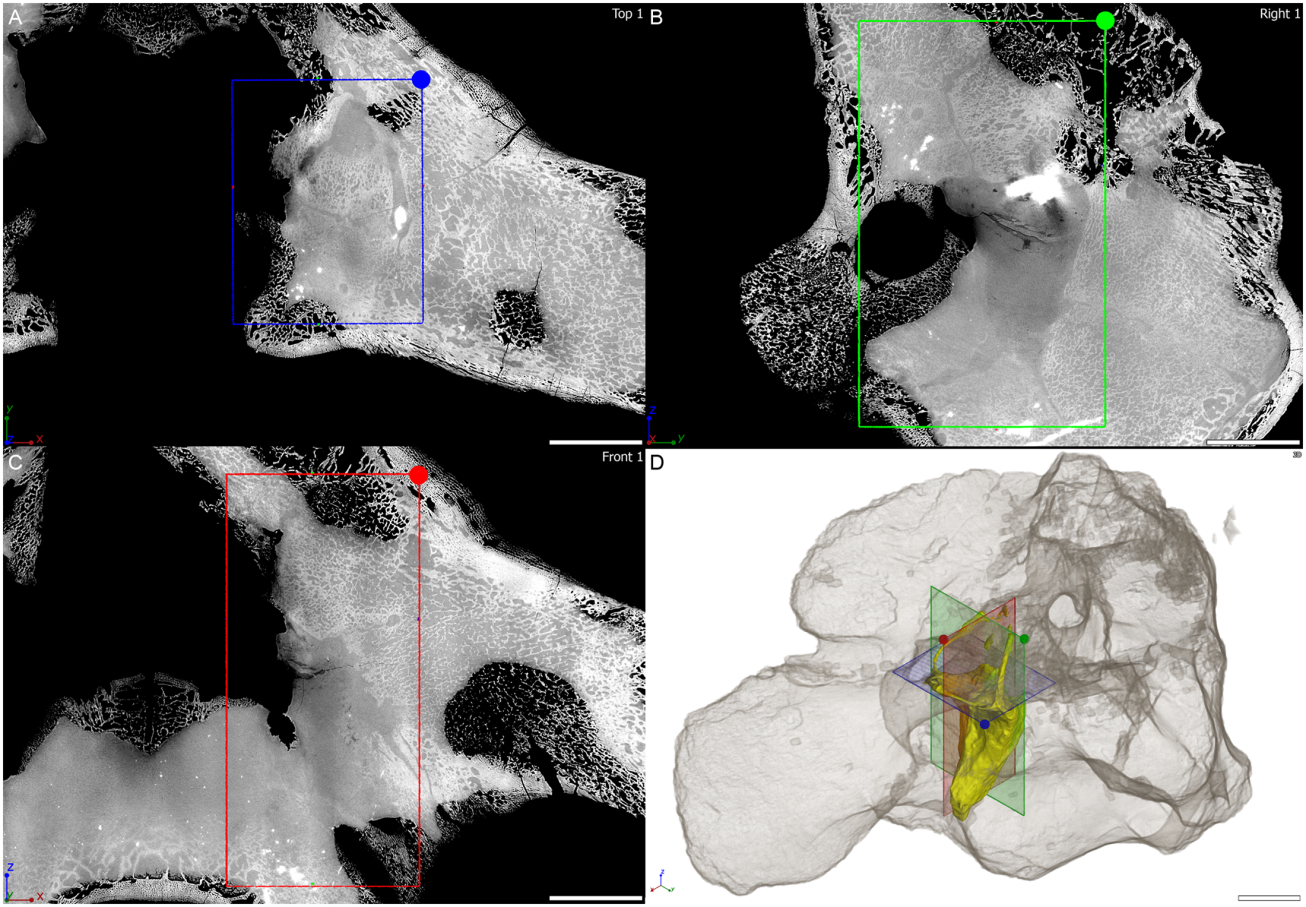
Virtual thin section through the osseous labyrinth of MTA/ACL001 specimen. Orthogonal virtual thin sections in A, horizontal plane, B, coronal plane, C, sagittal plane through the right osseous labyrinth of MTA/ACL001 specimen. D, three-dimensional rendering of the right osseous labyrinth with semi-transparent outline of the whole specimen in anterolaterodorsal view. Scale bar: 10 mm.

**Fig 5:**
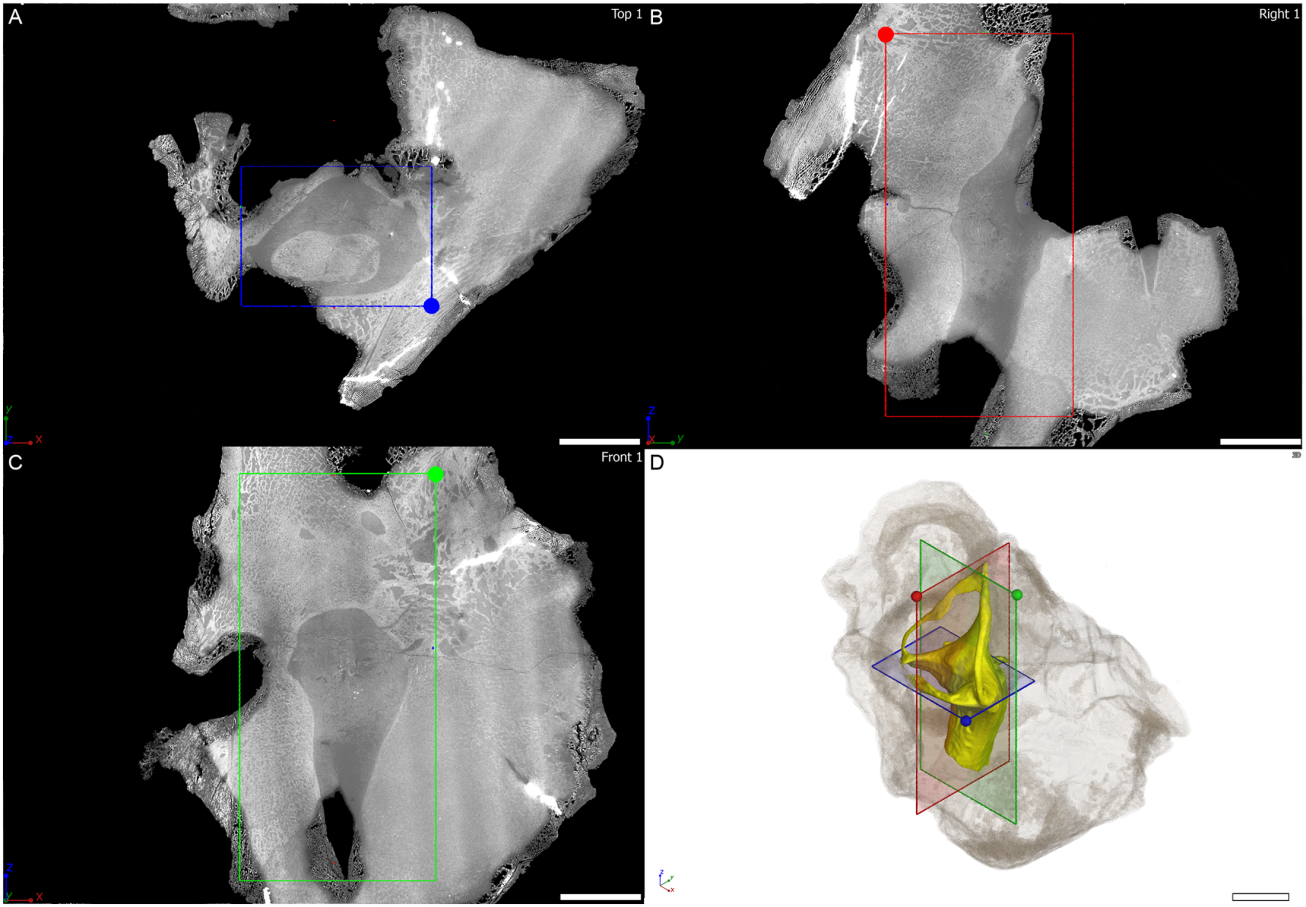
Virtual thin section through the osseous labyrinth of MTA/ACL002 specimen. Orthogonal virtual thin sections in A, horizontal plane, B, coronal plane, C, sagittal plane through the right osseous labyrinth of MTA/ACL002 specimen. D, three-dimensional rendering of the right osseous labyrinth with semi-transparent outline of the whole specimen in anterolaterodorsal view. Scale bar: 10 mm.

**Fig 6:**
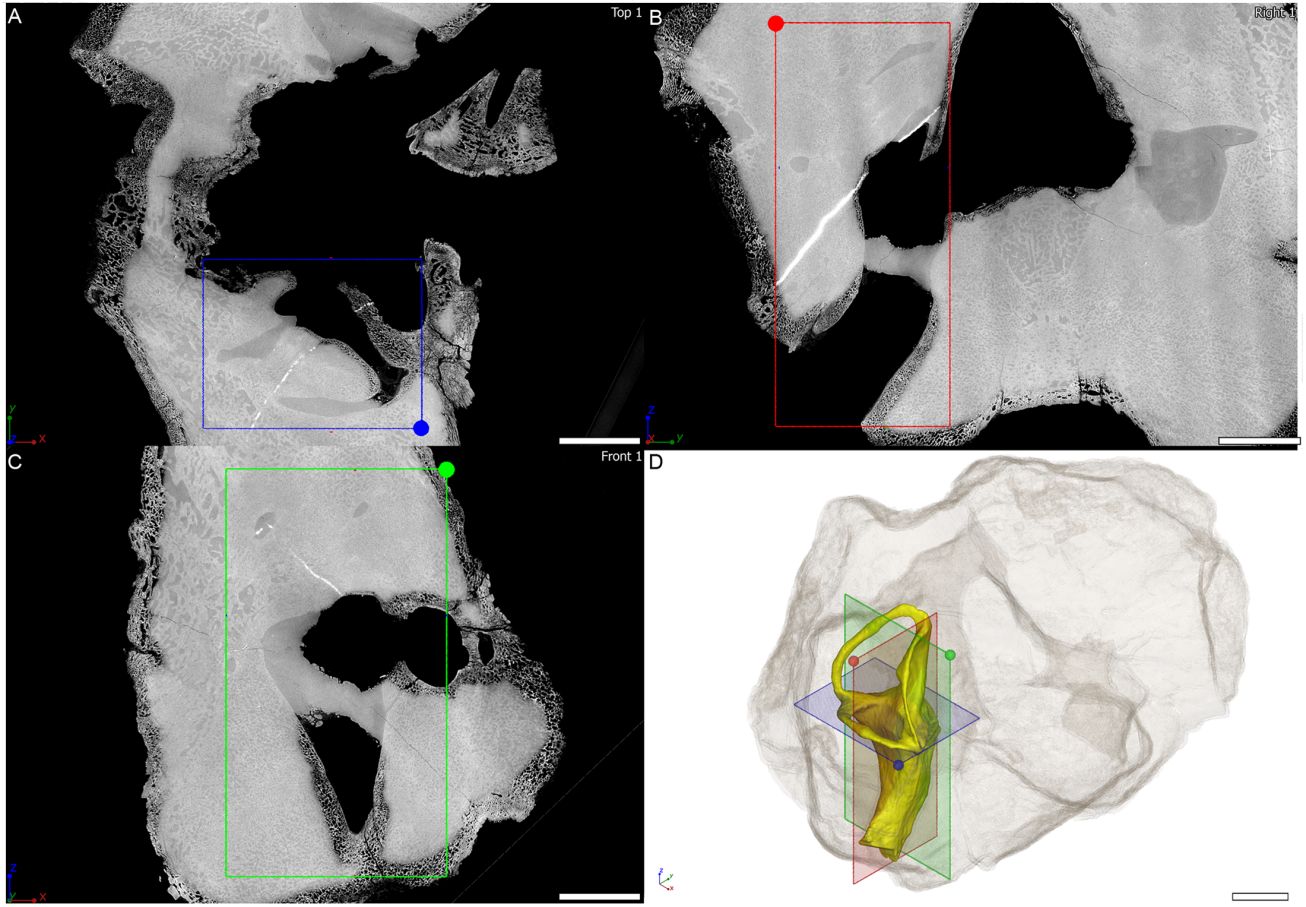
Virtual thin section through the osseous labyrinth of MTA/ACL003 specimen. Orthogonal virtual thin sections in A, horizontal plane, B, coronal plane, C, sagittal plane through the left osseous labyrinth of MTA/ACL003 specimen. D, three-dimensional rendering of the left osseous labyrinth with semi-transparent outline of the whole specimen in posterolaterodorsal view. Scale bar: 10 mm.

The *Endothiodon* specimens were scanned at the beamline ID17 of the European Synchrotron Radiation Facility (ESRF, Grenoble, France), using Propagation Phase Contrast Synchrotron micro-Computed Tomography (PPC-SRμCT). The set up consisted of a 130 keV monochromatic beam (bent double Laue), a tapered scintillating fiber-optic, a 0.5x set of lenses and a FReLoN-2K camera resulting in an isotropic voxel size of 45.98 μm in reconstructed data. The data acquisition comprised 3100 projections of 0.1 s each over 360°, and laterally shifted center of rotation to increase the reconstructed horizontal field of view (i.e., half-acquisition protocol [46]). Consecutive scans had an approximate 35% vertical overlap to compensate for the vertical beam profile. To produce Figs 4, 5 and 6 three orthogonal virtual thin sections are oriented based on the plane containing the horizontal semi-circular canal. All virtual thin sections result from the maximum intensity projection of three adjacent tomograms in order to reduce the density gradient present near the edge of the specimen. The contrast on the images has been adjusted setting the background to 0 and reaching saturation for the denser inclusion in the specimen.

The *Niassodon* specimen was imaged at the ID19 beamline of the European Synchrotron Radiation Facility (ESRF, Grenoble, France). The setup consisted of a FReLoN-2k camera, a 0.475x magnification set of lenses, a 750 μm LuAG scintillator, white beam from a W150 wiggler (gap 58 mm) filtered with Al 2 mm and Cu 4 mm (detected total integrated energy at 97.8 keV) and a sample-detector distance of 16 m to perform Propagation Phase Contrast Synchrotron micro Computed Tomography (PPC-SRμCT). The tomography was computed based on 6000 projections of 0.1 s each over 360 degrees resulting in data with a 27.85 μm isotropic voxel size. Additionally, the center of rotation was shifted by ~18 mm to increase the horizontal field of view in the reconstructed data (i.e., half acquisition protocol).

Both tomographic reconstructions were performed using the single distance phase retrieval approach of the software PyHST2 [47,48]. For this purpose, a range of ð/ß values were tested first (500–2000, steps of 250) from which a value of 1000 was selected as it was giving best contrast on reconstructed slices. The resulting 32-bit data were converted to a stack of 16-bit tiff images using the minimum and maximum values excluding 0.001% of voxels on both sides of the 3D histogram generated by PyHST2.

Volume processing and rendering was undertaken using the software VGstudio MAX 2.1 (Volume Graphics, Heidelberg, Germany). The segmentation was carried out using semi-automatic 3D region growing tools. When this tool did not permit complete extraction (e.g., contrast too low between the bone and the matrix or elevated fracture level), missing parts were added slice by slice using manual segmentation as necessary.

### Data accessibility

The tomographies described here are made accessible as.jpeg2000 stacks through the ESRF Paleontological Database (paleo.esrf.eu).

### Measurements and statistics

**Fig 7:**
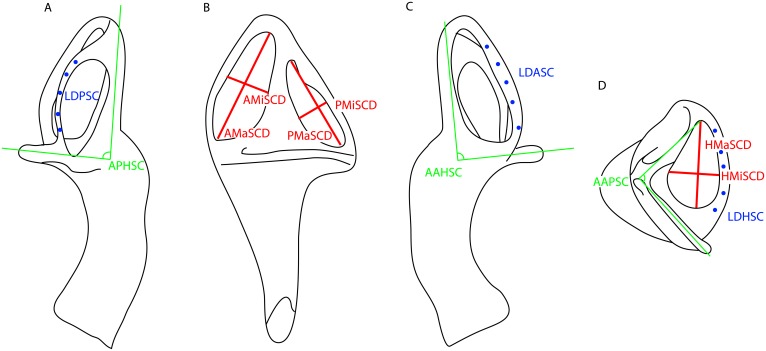
Diagrammatic scheme of the linear measurements performed. Osseous labyrinth in **A**, posterior view. **B**, lateral view. **C**, anterior view. **D**, dorsal view. Abbreviations: APHSC, angle between the posterior and horizontal semicircular canal, AAHSC, angle between the anterior and horizontal semicircular canal, AAPSC, angle between the anterior and posterior semicircular canal. LDASC, lumen diameter of the anterior semicircular canal. LDPSC, lumen diameter of the posterior semicircular canal. LDHSC, lumen diameter of the anterior semicircular canal. AMaSCD, anterior major axis of the semicircular canal diameter. AMiSCD, anterior minor axis of the semicircular canal diameter. PMaSCD, posterior major axis of the semicircular canal diameter. PMiSCD, posterior minor axis of the semicircular canal diameter. HMaSCD, horizontal major axis of the semicircular canal diameter. HMiSCD, horizontal minor axis of the semicircular canal diameter.

The measurements were performed in Amira (FEI, Hillsboro, Oregon, USA) using the 2D angle measurement tool and the 3D linear measurement tool following the scheme of Fig 7 (Tables 1, 2, 3, S1 and S2 Tables). To calculate the elliptical eccentricity of the vertical semicircular canals we used the formula $e=\sqrt{1-{\left(a/b\right)}^{2}}$, where *a* is the major axis, and *b* the minor axis of the SC. We used the surface thickness computation module in Amira to perform the lumen diameter measurements of the various semicircular canals. Each measurement was performed five times at different occasions to ensure repeatability and to test intra-observer variability (Tables 2, 3, S1 and S2 Tables). The five measurements of lumen diameter were performed in relatively equidistant points along the semicircular canal. Summary statistics were calculated for the linear measurements of the lumen diameter of the SCs (Table 2), whereas circular statistics [49] were used to treat the angular measurements between the three SCs for each specimen (Table 3). We used the t-distribution with two tails and n-1 degrees of freedom. We also calculated the relative and absolute Technical Error of Measurement (TEM) and performed repeated measures ANOVA’s for the angles between SC’s because these are truly repeated measurements (S2 Table); the lumen diameters are not made in the same homologous points (see Fig 7). Therefore, variations through the SCC are expected, so that the TEM cannot be calculated. Also, because the TEM is designed for 2 repeated measurements, not 5, we calculated the TEM for all pairwise combinations between the 5 repeated measurements and then averaged the calculated TEM for all 10 pairwise combinations.

**Table 1:** Vertical semicircular canal elliptical eccentricity measurements for the mammalian sampled taxa, the *Endothiodon* specimens and *Niassodon*. Abbreviations: AMiSCD, anterior semicircular canal minor axis diameter, AMaSCD, anterior semicircular canal major axis diameter, PMiSCD, posterior semicircular canal minor axis diameter, PMaSCD, posterior semicircular canal major axis diameter, HMiSCD, horizontal semicircular canal minor axis diameter, HMaSCD, horizontal semicircular canal major axis diameter, LogBM, log10 body mass, asterisk refers to estimated body mass calculated using the linear regression model. See also S3 Table.

Genus	Specimen	AMaSCD	AMiSCD	PMaSCD	PMiSCD	HMaSCD	HMiSCD	Log10 BM	*e*
*Atelerix*	unvouchered	1.33	1.22	1.23	1.08	0.96	0.89	2.52	0.44
*Canis*	TMMM-150	1.73	1.72	1.42	1.42	1.50	1.46	4.60	0.10
*Cavia*	TMM-M-7283	2.15	1.53	1.46	1.44	1.90	1.16	2.86	0.57
*Chrysochloris*	AMNH82372	1.20	0.78	0.68	0.48	0.67	0.58	1.67	0.75
*Cynocephalus*	AMNH187859	2.09	1.95	1.64	1.53	1.49	1.37	3.10	0.36
*Dasypus*	TMM-M-152	1.90	1.50	1.63	1.62	1.45	1.38	3.60	0.47
*Dasypus*	TMM-M-1065	1.96	1.66	1.80	1.72	1.44	1.40	3.60	0.44
*Dasypus*	TMM-M-1880	2.06	1.31	1.88	1.72	1.58	1.54	3.60	0.64
*Dasypus*	TMM-M-1885	2.15	1.31	1.94	1.93	1.48	1.37	3.60	0.61
*Didelphis*	TMM-M-2517	1.47	1.40	1.11	1.10	0.92	0.84	3.39	0.25
*Equus*	TMM-M-171	3.53	3.43	3.49	3.20	3.10	3.04	5.61	0.33
*Eumetopias*	unvouchered	3.32	2.38	2.83	2.32	2.59	1.91	5.58	0.64
*Felis*	TMM-M-968	1.99	1.67	1.84	1.73	1.73	1.53	3.46	0.46
*Hemicentetes*	AMNH161535	1.13	0.95	0.79	0.65	0.69	0.58	2.13	0.55
*Homo*	UTO-HS01	1.99	1.83	2.83	2.38	2.72	2.51	4.77	0.49
*Macaca*	TMM-M-5987	2.68	1.34	2.36	2.27	2.36	2.02	5.67	0.70
*Macroscelides*	AMNH161535	1.19	1.16	1.07	0.73	1.17	0.76	1.59	0.55
*Manis*	AMNH53896	1.40	1.13	1.40	1.15	0.82	0.78	3.19	0.58
*Monodelphis*	TMM-M-7599	1.02	1.00	0.94	0.87	0.74	0.69	1.97	0.29
*Mus*	TMM-M-3196	0.86	0.58	0.71	0.47	0.59	0.52	1.29	0.74
*Nycteris*	AMNH268369	0.99	0.85	0.81	0.70	0.96	0.61	1.47	0.50
*Orycteropus*	AMNH51909	3.36	2.86	4.07	2.95	3.44	2.88	4.75	0.62
*Procavia*	TMM-M-4351	2.15	1.57	2.30	1.72	2.03	1.48	3.47	0.67
*Pteropus*	AMNH237593	1.54	1.36	1.45	1.15	1.28	1.25	2.50	0.54
*Rhinolophus*	AMNH245591	0.87	0.70	0.74	0.66	0.92	0.77	1.35	0.53
*Sorex*	unvouchered	0.78	0.41	0.70	0.43	0.44	0.44	0.84	0.83
*Sus*	TMM-M-2689	2.46	2.00	2.20	1.53	1.88	1.56	4.93	0.65
*Sylvilagus*	TMM-M-2689	1.75	1.69	1.42	1.28	1.27	1.18	3.08	0.34
*Tadarida*	TMM-M-3030	0.83	0.73	0.69	0.61	0.89	0.61	1.10	0.48
*Trichechus*	MSW03156	4.07	4.06	3.72	3.59	4.53	4.27	5.67	0.19
*Tupaia*	TMM-M-2256	1.91	1.61	1.67	1.26	1.84	1.20	2.12	0.60
*Tursiops*	SDNHM21212	1.08	0.96	0.80	0.68	1.29	1.27	5.45	0.49
*Endothiodon*	MTA-ACL-002	5.24	2.00	4.61	1.81	3.24	1.74	5.15*	0.92
*Endothiodon*	MTA-ACL-003	4.93	2.74	3.91	1.87	2.97	1.62	5.06*	0.85
*Endothiodon*	MTA-ACL-001	5.15	2.64	4.24	1.96	3.47	2.09	5.26*	0.87
*Niassodon*	ML1620	2.4	1.68	2.08	1.08	3.46	2.93	2.69*	0.79

**Table 2:** Linear measurements for each *Endothiodon* cf. *bathystoma* specimen. Each variable for the three specimens was measured at five different loci of each SC. Abbreviations: SE, standard error, σ, variance, conf. int. confidence interval, LDASC Lumen diameter of the anterior semicircular canal, LDPSC Lumen diameter of the posterior semicircular canal, LDHSC Lumen diameter of the horizontal semicircular canal.

	LDASC	LDPSC	LDHSC
MTA-ACL-001	0.75	0.42	0.57
	0.80	0.35	0.58
	0.80	0.40	1.02
	0.84	0.42	0.88
	0.76	0.39	0.66
Mean	0.79	0.40	0.74
σ	0.04	0.03	0.20
St error	0.02	0.01	0.09
Lower bound 95% conf. int.	0.75	0.36	0.49
Upper bound 95% conf. int.	0.83	0.43	0.99
MTA-ACL-002	0.69	0.43	0.76
	0.81	0.38	0.77
	0.94	0.44	0.73
	0.59	0.44	0.88
	0.53	0.42	0.66
Mean	0.71	0.42	0.76
σ	0.17	0.02	0.08
St error	0.07	0.01	0.04
Lower bound 95% conf. int.	0.51	0.39	0.66
Upper bound 95% conf. int.	0.92	0.45	0.86
MTA-ACL-003	0.61	0.41	0.41
	0.68	0.36	0.62
	0.64	0.40	0.47
	0.70	0.42	0.77
	0.71	0.41	0.51
Mean	0.67	0.40	0.56
σ	0.04	0.02	0.14
St error	0.02	0.01	0.06
Lower bound 95% conf. int.	0.62	0.37	0.38
Upper bound 95% conf. int.	0.72	0.43	0.73

**Table 3:** Angular measurements and respective descriptive circular statistics for each *Endothiodon* cf. *bathystoma* specimen. Five repeated measurements of the angle between the three SCs was measured for the three sampled specimens. Abbreviations: conf. int., confidence interval, AAHSC, Angle between the anterior and horizontal semicircular canal, APHSC, Angle between the posterior and horizontal semicircular canal, AAPSC, Angle between the anterior and posterior semicircular canal.

	AAHSC	APHSC	AAPSC
MTA-ACL-001	91.7	90.4	89.7
	92	90.5	89.4
	90.6	90.1	88.8
	91.5	89.2	89.6
	89.6	90.1	90.9
Circular mean	91.08	90.06	89.68
Circular standard deviation	0.84	0.25	0.37
Standard error	0.38	0.11	0.16
Lower bound 95% conf int	90.03	89.75	89.23
Upper bound 95% conf int	92.13	90.37	90.13
MTA-ACL-002	91.2	91.7	88.6
	92.2	91.2	90.1
	92.2	92.0	89
	92.5	89.2	89.0
	92.1	89.8	89.7
Circular mean	92.04	90.78	89.28
Circular standard deviation	1.8	0.67	0.66
Standard error	0.8	0.3	0.29
Lower bound 95% conf int	89.81	89.95	88.46
Upper bound 95% conf int	94.27	91.61	90.1
MTA-ACL-003	90.5	89.5	87
	91.4	90.4	88
	91.5	90.2	90.3
	90.7	90.4	90.4
	88.9	89.6	89.6
Circular mean	90.08	89.91	87.44
Circular standard deviation	91.12	90.13	90.68
Standard error	0.19	0.04	0.58
Lower bound 95% conf int	90.22	89.94	87.89
Upper bound 95% conf int	90.98	90.1	90.23

## Results

### Anatomical description of the *Endothiodon* bony labyrinth

**Fig 8:**
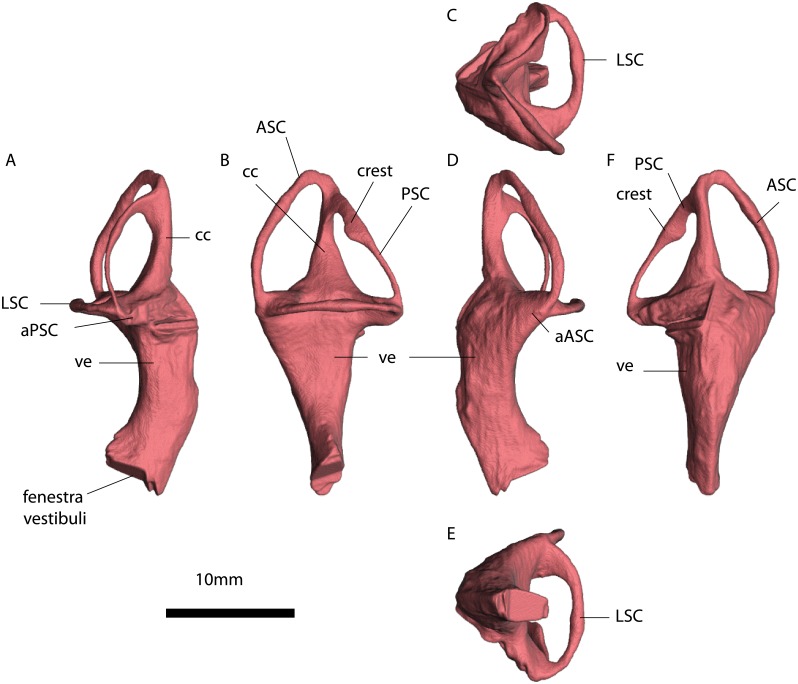
MTA/ACL001 left inner ear. A, anterior view. B, lateral view. C, dorsal view. D, anterior view. E, ventral view. F, medial view. Abbreviations: ASC, Anterior semicircular canal. PSC, Posterior semicircular canal. HSC, horizontal semicircular canal. ve, vestibulus. cc, crus communis. aASC, Ampulla of the anterior semicircular canal. aPSC, Ampulla of the posterior semicircular canal, “*”, estimates based on the average radius of the semicircular canal. See also S1 Fig.

**Fig 9:**
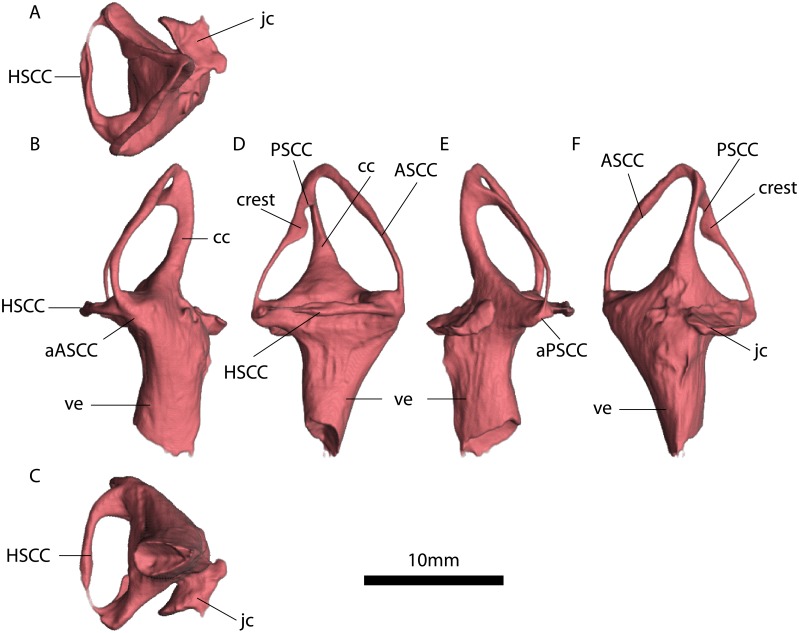
MTA/ACL002 right inner ear. A, dorsal view. B, anterior view. C, ventral view. D, lateral view. E, posterior view. F, medial view. Abbreviations: ASCC, Anterior semicircular canal. PSCC, Posterior semicircular canal. HSCC horizontal semicircular canal. ve. vestibulus. cc. crus communis. aASCC, Ampulla of the anterior semicircular canal. aPSCC, Ampulla of the posterior semicircular canal. jc. jugular canal. See also S1 Fig.

**Fig 10:**
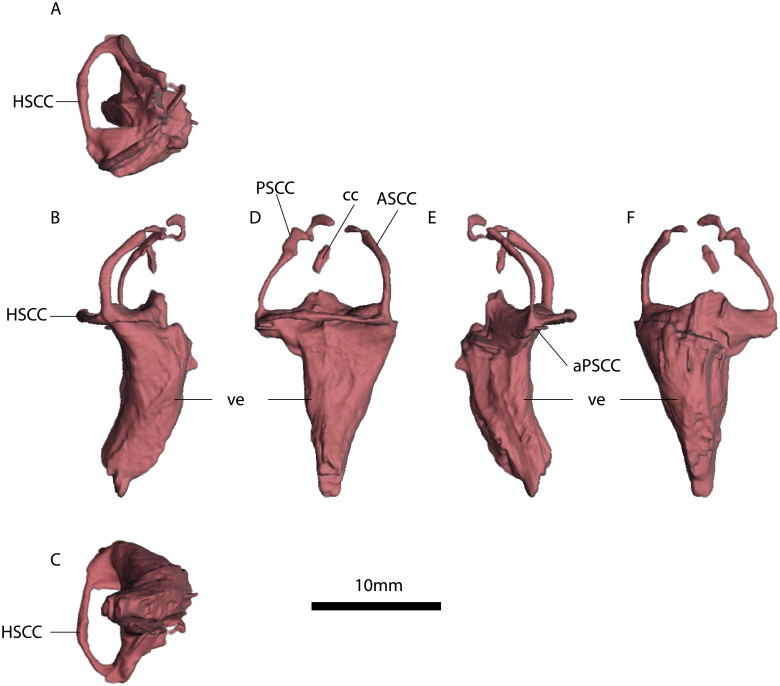
MTA/ACL003 right inner ear. A, dorsal view. B, posterior view. C, ventral view. D, lateral view. E, anterior view. F, medial view. Abbreviations: ASCC, Anterior semicircular canal. PSCC, Posterior semicircular canal. HSCC, Horizontal semicircular canal. ve, vestibulus. cc, crus communis. aPSCC, Ampulla of the posterior semicircular canal. See also S3 Fig.

The *Endothiodon* osseous labyrinths are exquisitely preserved (Figs 8, 9, 10, S1, S2 and S3 Figs) except for small dorsal portions of the anterior and posterior SCs as well as a part of the crus communis in MTA/ACL003 (Fig 10).

**Fig 11:**
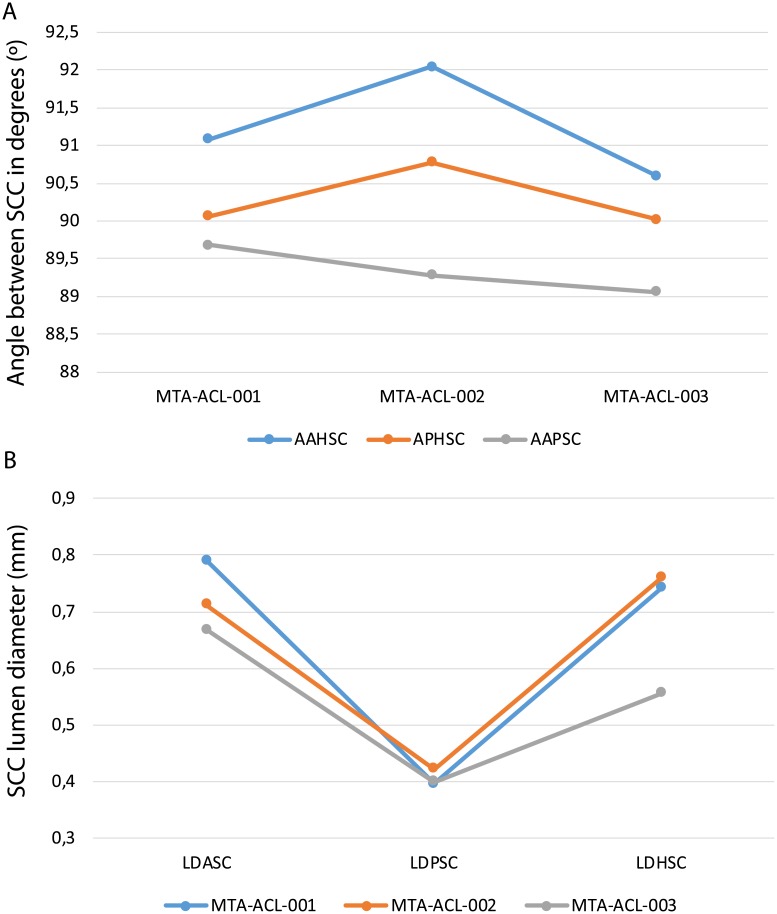
Comparative charts between the various semicircular canals among the different *Endothiodon* cf. *bathystoma* specimens. A, angle between the semicircular canals. B, lumen diameter of the various semicircular canals. Notice that all the semicircular canals are nearly orthogonal, but the AAHSC is consistently greater than 90°, and the AAPSC is consistently lower than 90°. Also, the horizontal and the anterior semicircular canals have consistently similar lumen diameters and the posterior semicircular canal is consistently the thinnest.

**Fig 12:**
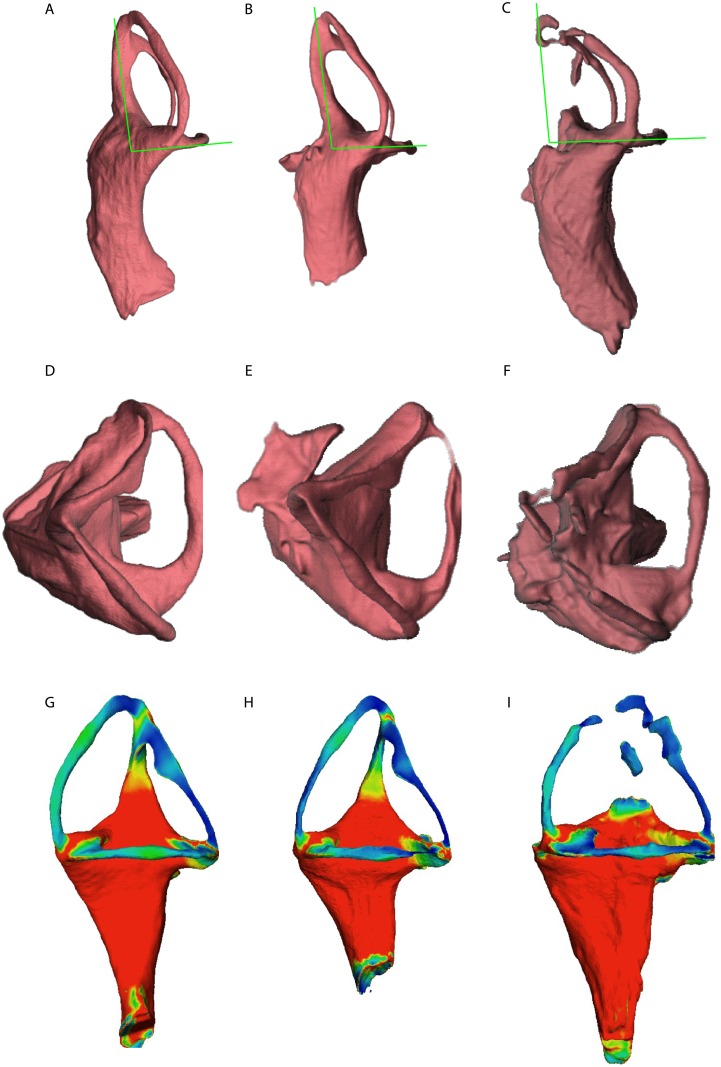
Comparison between the three *Endothiodon* cf. *bathystoma* specimens. Images are not at scale to facilitate comparisons. A, MTA-ACL001 in anterior view. B, MTA-ACL002 in anterior view (reversed). C, MTA-ACL003 in anterior view (reversed). D, MTA-ACL001 in dorsal view. E, MTA-ACL002 in dorsal view (reversed). F, MTA-ACL003 in dorsal view (reversed). Variation of semicircular canal lumen diameter for: G, MTA-ACL001 in lateral view, H, MTA-ACL002 in lateral view (reversed), I, MTA-ACL003 in lateral view (reversed).

The fenestrae vestibuli and lagena are not clearly distinguishable. The bone surrounding the fenestra vestibuli is not completely preserved, missing the lateralmost portions of the basisphenoid and opisthotic. The vestibule is slightly curved, with a medial curvature convexity (Figs 8, 9 and 10, S1, S2 and S3 Figs). The vestibule is a broad chamber, which is subtriangular in cross section. It develops the cochlea ventrally as a straight canal. The major axis radius of the anterior SC is slightly longer (average = 9.47 mm) than the posterior SC (average = 8.47 mm); see Table 1. The anterior SC major axis is 1.37 to 2.49 times longer than the minor axis, whereas the posterior SC is slightly more eccentric (2.20 to 2.56; Table 1). At the level of the fenestra vestibuli a horizontal SC develops laterally. The horizontal SC is ellipsoidal in shape, with a major axis ranging from 8.03 to 8.48 mm, and the minor axis from 3.05 to 3.50 mm (Figs 8, 9 and 10). The horizontal SC is broad near the vestibulum but it becomes abruptly thin laterally. Similarly, the vertical SCs are thin and dorsoventrally elongated. The SCs are suborthogonally-oriented relative to each other (Table 3, Figs 11, 12). However, the angle between the anterior and horizontal SC is consistently around ~91° in the different specimens, and the angle between the anterior and posterior SC is consistently around ~89° (Fig 12A).

The lumina of the SCs are subcircular (i.e., elliptical eccentricity less than 0.15) to slightly elliptical in cross-section. Interestingly, the posterior SC possesses a crest close to the crus communis (Figs 8 and 9). The narrowest duct radius of the anterior SC ranges from 0.67 to 0.79 mm, and for the posterior SC from 0.40 to 0.42 mm (Table 2). The lumen diameter of the anterior and horizontal SCs are nearly the same (0.6–0.8 mm); however, the lumen of the posterior SC is consistently thinner (~0.4 mm; Fig 11B). Additionally, in dorsal view, the posterior SC is significantly more arched anteriorly than the horizontal and anterior SCs. The horizontal and anterior SCs are in-plane, i.e., there are no significant deviations from a planar toroid (Fig 12D, 12E and 12F). The osseous enclosure around the ampullae in all three SCs are poorly distinguishable from the slender portion of the canal. At the intersection of the anterior and horizontal SC there is an inflated portion, elliptical in cross section, which forms the secondary crus communis and houses the ampulla. The osseous enclosure around the ampulla of the posterior SC is slightly expanded ventrally, but also confounds with the posterior intersection of the horizontal SC. The crus communis is very broad at the base but becomes precipitously thin towards its dorsal portion, giving a subtriangular aspect. The cross-section of the crus communis changes dorsoventrally, being D-shaped ventrally with the convexity being laterally-oriented, subcircular at the midpoint and then becoming ellipsoidal dorsally. The jugular (vagal) canal meets the osseous labyrinth near the base of the posterior SC.

### Anatomical description of the *Niassodon* bony labyrinth

**Fig 13:**
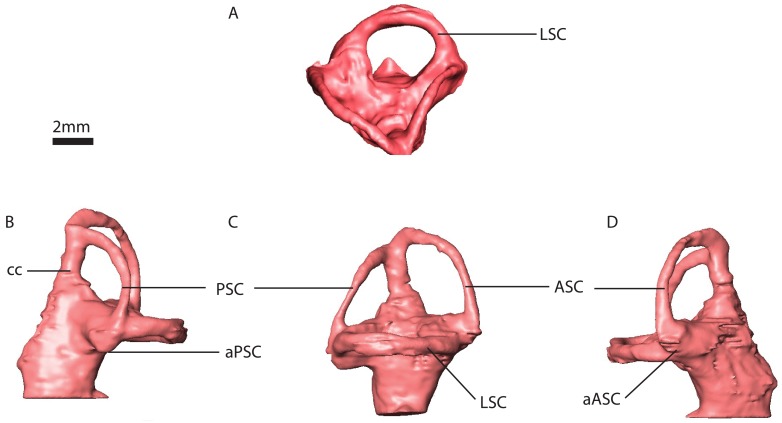
*Niassodon mfumukasi* (ML 1620) right inner ear. A, dorsal view. B, posterior view. C, lateral view. D, anterior view. Abbreviations: ASC, Anterior semicircular canal. PSC, Posterior semicircular canal. LSC, horizontal semicircular canal. aPSC, ampulla posterior semicircular canal. aASC, ampulla anterior semicircular canal. cc, crus communis.

Based on new segmentation derived from a PPC-SRμCT scan, we provide further details on the anatomy of *Niassodon* inner ear (Fig 13), a pivotal taxon related to *Endothiodon* according to the most recent phylogenetic analysis [50,51]. Indeed, the small-bodied *Niassodon* possesses relatively elongated SCs [35]. In *Niassodon*, the vestibular system is delimited by the supraoccipital, prootic and opisthotic. The supraoccipital delimits the posterior SC, the crus communis except its base and the posterior third of the anterior vertical SC. The prootic envelops the anterior two-thirds of the anterior vertical SC, the anterior part of the vestibule and the anterior portion of the horizontal SC, and the base of the crus communis. The lagena is delimited by the exoccipital, basisphenoid and basioccipital. The exoccipital envelops the posterior portion of the vestibule and a small dorsomedial portion of the lagena. The medial and posterior part of the lagena, as well as the dorsal part of the lateral aspect of the fenestra vestibuli is delimited by the basisphenoid. The basioccipital delimits the posterior aspect of the lagena.

The SCs are ellipsoidal and orthogonally oriented with respect to each other (Fig 13). The anterior SC projects higher dorsally than the posterior SC. The least eccentric is the horizontal SC with 0.5 mm difference between the major and minor axis on average (Table 1). On the other hand, the posterior SC is the most eccentric with a 1.9 mm difference between major and minor axis (Table 1). The anterior SC is two and a half times more eccentric (1.25 mm) than the horizontal SC (Table 1).

Measured at the thinnest section of the canal, the horizontal SC is the thickest among the three with a diameter of averaging 0.61(±0.01) mm (Fig 13). The posterior SC is the thinnest (0.34±0.03 mm) and the anterior SC is about 0.47 mm diameter. Midway on the left anterior SC there seems to be a constriction (0.24 mm cross-section), but it appears to be an artifact of segmentation. The crus communis is nearly twice as thick as the vertical SCs, straight but somewhat expanded at the base (Fig 13).

The anterior ampulla is a projecting globular structure, detaching from the vestibule (Fig 13). The posterior ampulla is integrated with the vestibule, yet forming a ventral projection giving a somewhat rectangular appearance in posterolateral view. The ampulla of the horizontal canal cannot be differentiated from the vestibule (Fig 13).

The lagena projects ventrally from the SC system but it is truncated along the sagittal plane (Fig 13). Dorsally, near the vestibule, the lagena is a stout D-shaped cylinder with the apex of the curvature projecting posterolaterally, then the lagena tapers ventrally to an acute tip.

### Intraspecific variation

The summary statistics for all of the morphometric variables allow us to understand measurement precision and by comparing the three specimens we have some information on intraspecific variation despite the small sample size. The lumen diameter of the anterior SC varies between specimens from 0.67–079 mm (we used the mean of the repeated measures for each variable to calculate the sample range) with a mean of 0.72 mm, likewise for the posterior SC varies from 0.40–0.42 mm averaging 0.41 mm, and the horizontal SC varies from 0.56–0.76 mm averaging 0.69 mm. The standard error for the lumen diameter of the anterior SC 0.03 mm, for the posterior SC 0.01 mm, and the horizontal SC 0.04 mm.

The angle between the horizontal and posterior SCs ranges from 90.02 to 90.78° averaging 90.29°, between the horizontal and anterior SCs ranges from 90.6 to 92.04° averaging 91.24°, and the angle between the anterior and posterior SCs ranges from 89.06 to 89.68° averaging 89.34°. The angle between the horizontal and posterior SCs has a standard error of 0.25°, for the angle between the horizontal and anterior SCs 0.18°, between the horizontal and posterior SCs is 0.25° and between the anterior and posterior SCs is 0.31°.

The repeated measures of the linear variables allow to check intra-canal variability. For the linear measurements, the standard error ranges from 0.01 mm for the posterior SC lumina diameters of all specimens and 0.09 mm for the lumina diameter of the horizontal SC in MTA/ACL001. Thus, linear measurements of the lumina diameters are relatively precise with errors not exceeding 14% of the measured quantities (Table 1). For the angular measurements, the repeated measurements allow to understand intra-observer variability. The average relative TEM is 0.82% for the angle between the posterior and horizontal SCs, 0.88% for the angle between the anterior and horizontal SCs, and 1.08% for the angle between the anterior and posterior SCs. Thus, angular measurements are very precise with errors not exceeding 1.08% of the measured quantities (Table 2, S2 Table). The overall average between angles for all SCs in all specimens is 90.29°.

### Body mass estimation

**Fig 14:**
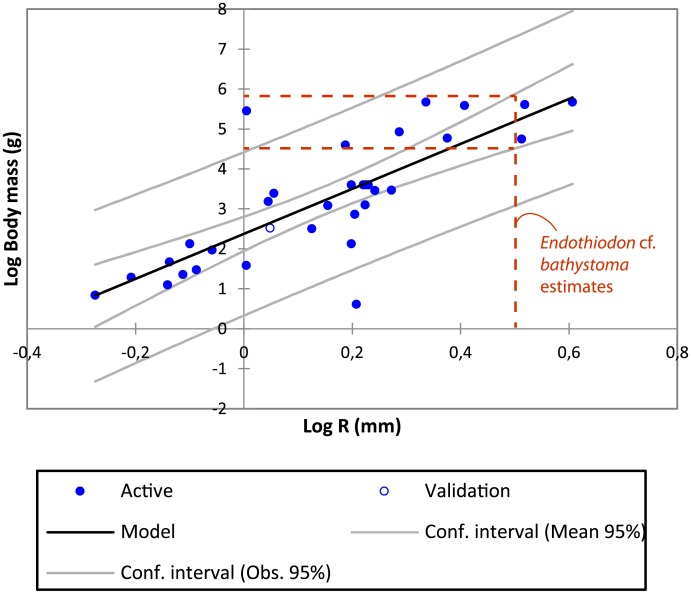
Linear regression of the log_10_ average semicircular canal radius to estimate the log_10_ body mass. The estimates for the *Endothiodon* cf. *bathystoma* specimens range from ~116 to 182 kg. The correlation coefficient between the two variables is 0.787. The extant mammal measurements can be found in S3 Table and from [15].

We used the average of all SC radii to estimate *Endothiodon* body mass (Fig 14), which has given statistically significant results by previous authors for other taxa [12,15]. The resulting linear regression equation, optimized for Amemiya Prediction Criterion, is highly significant (correlation coefficient = 0.787, ${\overset{-}{r}}^{2}=0.606$, P<0.0001, α = 0.05, see Fig 14). Significant deviations of the SC radius have so far only been detected in highly specialized taxa (e.g., [12]). The resulting linear regression equation estimates that *Endothiodon* weighed between ~116 to 182 kg. MTA/ACL002 is the *Endothiodon* specimen closest to the average (140 kg).

## Discussion

### Intraspecific variation of the semicircular canal system

The summary statistics on the *Endothiodon* cf. *bathystoma* angular and linear measurements indicate low intraspecific variability, despite the small sample. However, as there is currently limited information on dicynodont inner ear morphology in the literature, we cannot conclude at this stage whether low intraspecific variability is a common trend among dicynodonts. It is crucial to investigate, when possible, more than one specimen of the same dicynodont taxon. High intraspecific variability in the vestibular system has been linked to relaxed selective pressures resulting, for instance, from slow and infrequent movements [40]. However, although our results tend to suggest that *Endothiodon* cf. *bathystoma* was not a slow-moving organism, we cannot perform the Levene’s heteroscedasticity test due to the small sample size. In addition to this possible low variability in the SCs of *Endothiodon* cf. *bathystoma*, we found several other conservative morphological characters among specimens (see below).

Our results show that the SCs of *Endothiodon* are nearly orthogonal, as the sum of the squared differences from orthogonality for all specimens only amounts to 7.8 (Table 3, S2 Table). Malinzak et al. [4] suggest that small deviations from SC orthogonality are associated with agility in strepsirrhine primates, and this rationale has been applied to various other mammalian synapsids (e.g., [21]). However, it is most likely that *Endothiodon* was not as agile as an arboreal primate, but that instead performed rapid head movements, which would also explain our results.

The pig, *Sus*, which are animals of comparable body mass to *Endothiodon*, do fast head movements while foraging and ingesting food [52] and have elevated SC eccentricity (*e*~0.65) and near orthogonal SCs. *Sus* SCs are quasi-orthogonal too (AAHSC is 86°, APHSC is 89.7° and AAPSC is 90.8°). *Endothiodon* could have employed a similar foraging and food processing behavior. Based on the occipital index, previous authors [53] suggest that *Endothiodon* performed a significant degree of lateral head movements, and the evidence here provided is consistent with such behavior.

### Comparative anatomy and evolution of Endothiodontia inner ears

*Niassodon* has been recovered as the sister-taxon of *Endothiodon* in recent phylogenetic analyses [50,51], and hence comparisons between the taxa are relevant, as they both belong to Endothiodontia. The vertical SCs of *Endothiodon* (n = 3) are substantially more eccentric (*e*~0.88) than those of *Niassodon* (*e*~0.79, n = 1). Additionally, the anterior and posterior SCs of *Endothiodon* are more vertically positioned than those of *Niassodon*. In *Niassodon* the SCs are elliptical but rotated sub-horizontally whereas in *Endothiodon* they are vertically oriented (Figs 8–10 and 13). These results suggest that within the Endothiodontia lineage there was a re-orientation and elongation of the vertical SCs, leading to changes in sensitivity of the vestibular system [5,45]. The surprisingly eccentric *Endothiodon* SCs may be related to the specialized feeding apparatus of this genus [53], which could require particular head movements for foraging, processing or ingesting food. Based on the anatomy of the oral apparatus, Cox and Angielczyk [53] described the food processing cycle of *Endothiodon* in detail and demonstrated that it was capable of cropping food items with a uniquely peculiar ‘hare lip’. Furthermore, the occipital region suggests *Endothiodon* had a significant degree of lateral head movements [53]. Despite the differences between the SCs of *Endothiodon* and *Niassodon*, both taxa have strikingly higher SC eccentricity than other known dicynodonts [27–38]. However, whereas this feature is unusual in the mammalian lineage (e.g., [15, 45]), it is premature to conclude that SC eccentricity was widespread among dicynodonts. Some authors [54] have speculated that the elongation of the anterior SC in dinosaurs may be linked to bipedalism, because humans also have similar SC elongation [55]. It was also suggested that horizontal SC elongation could be related to quick and powerful neck lateroflexion in tyrannosaurs [54]. Nevertheless, these hypotheses remain untested with robust biomechanical modelling.

**Fig 15:**
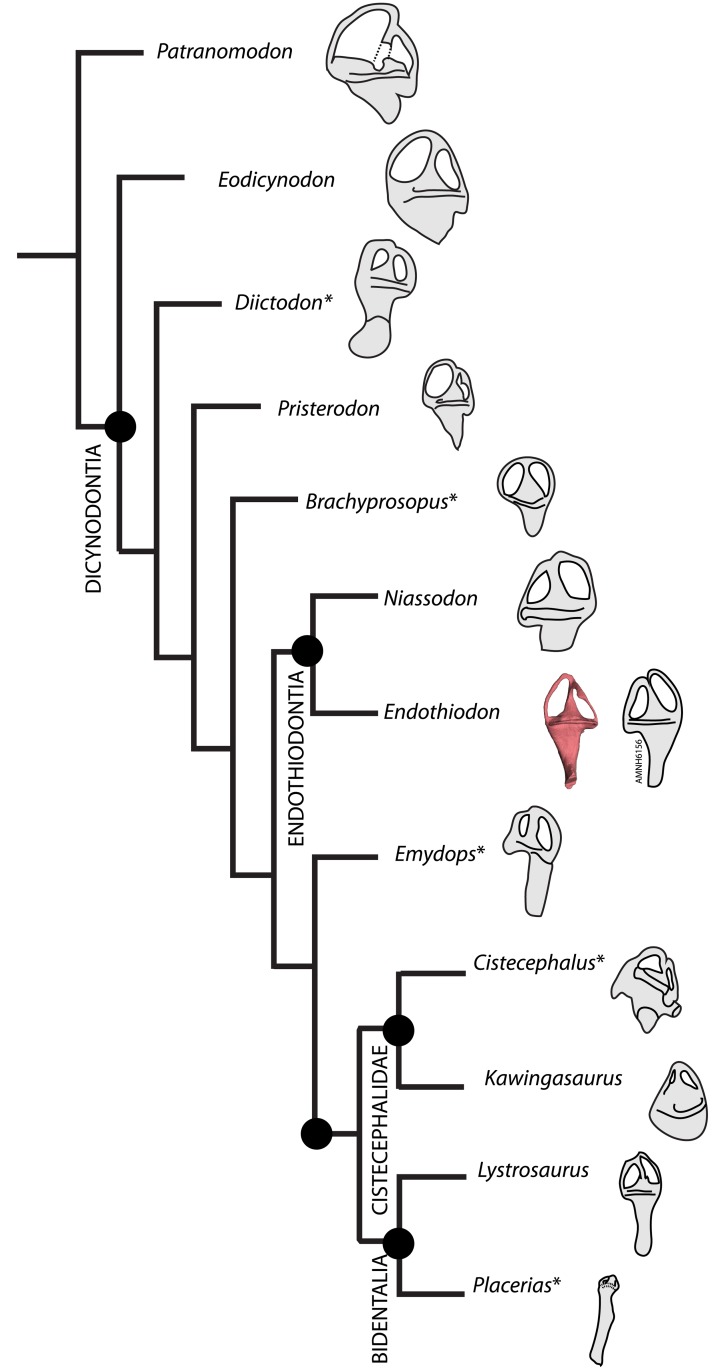
Simplified cladogram of published dicynodont bony labyrinths. Phylogeny based on the most recent analysis [51]. *Patranomodon*, *Eodicynodon*, *Pristerodon* and *Lystrosaurus* from [27]; *Diictodon* from [29]; *Brachyprosopus* from [38]; *Emydops* from [34]; *Cistecephalus* from [33]; *Kawingasaurus* from [35] and *Placerias* from [31]. Asterisks indicate bony labyrinths that did not result from computed tomography renderings.

The *Endothiodon* inner ear can be readily distinguished from that of *Eodicynodon* based on a posteriorly tilted crus communis (Fig 15). *Pristerodon* can be distinguished from *Endothiodon* because its horizontal SC is subcircular and the crus communis is not smoothly dorsally tapering (Fig 15). *Lystrosaurus* has a strongly ellipsoidal duct cross-section, contrary to the *Endothiodon* condition (Fig 15). Due to its inflated vestibule morphology [35], *Kawingasaurus* clearly contrasts with *Endothiodon* (Fig 15). Some features seem to be shared by other dicynodonts, such as a ventrally broad crus communis, a significantly shorter posterior SC compared to the anterior SC, the absence of well-delimited ampullae, and a certain degree of eccentricity of the SCs [27, 35] (Fig 15). However, *Endothiodon* seems to have considerably more eccentric canals than any other dicynodont published so far [27–38].

Other clades exhibit elevated eccentricity. For instance, caecilian amphibians have horizontally elongated SCs (e.g., [56]), squamates have obliquely-oriented elongated SCs (e.g., [7]), and some large-bodied dinosaurs have highly eccentric canals (e.g., [57, 58]). Additional studies outside Dicynodontia could elucidate broader evolutionary patterns of the SCs, namely a trend toward more circular canals across synapsid history, with some reversals in taxa with specific biomechanical demands, such as in Endothiodontia. Nevertheless, regardless of the possible direct causal functions for SC elongation, it appears that complex biomechanical stimuli (such as foraging habits, locomotion type, ecology, etc.) are related to the evolution of unusual eccentric morphologies.

Another important character in *Endothiodon* cf. *bathystoma* is the crest on the anterior SC, which is absent in *Niassodon*. This morphological feature has not been previously described and it is not an artifact of segmentation because it is consistently present in the individuals that could be measured (not in MTA/ACL003 because this section of the SCs is not preserved). Apart from these more striking differences, *Endothiodon* and *Niassodon* have relatively similar bony labyrinths. For instance, the SCs have a comparable development of the ampullae, both possess a triangular vestibule, the angles between SCs are similar, and the vertical SCs do not follow the same plane but are slightly deflected toward each other, particularly the posterior SC (see Anatomical Description). The current phylogenetic position of *Niassodon* and *Endothiodon* as sister taxa indicates that these characters are synapomorphies of Endothiodontia. Additional knowledge on the vestibular system of *E*. *tolani* [53] and *E*. *uniseries* [59] may reinforce these results. Based on the anatomical characters here provided and the natural cast of the AMNH6156 bony labyrinth, it seems that this specimen is an *Endothiodon* as well (S1 Text).

### *Endothiodon* body mass

Body mass is intimately related to various aspects of ecology and physiology [60–62]. In dicynodonts, body mass estimates are rare. We attempted to calculate body mass in *Endothiodon* based on SC dimensions, rather than skull length, because our specimens do not have complete skulls. Whereas several proxies based on specific skeletal elements such as postcranial bones have been used to estimate body mass in extinct taxa, body mass also has a significant correlation with SC dimension (e.g., [63]). We used an extant mammalian dataset [15] of osseous labyrinths as a baseline to calculate body mass estimates for the three *Endothiodon* specimens here described (Fig 14). Our results indicate that these *Endothiodon* specimens ranged between 116 kg and 182 kg.

SAM-PK-K11271 is a complete *Endothiodon* specimen whose propodial dimensions can be used to compare with the SC dimension estimates here provided. Other authors [64] derived equations to estimate body mass based on the femur and humerus circumference. SAM-PK-K11271 has a humeral and femoral narrowest midshaft circumference of 17.5 cm and 13.0 cm, respectively. Body mass estimates based on these estimates range from ~309 to 556 kg, using Campione and Evans equations [64]. In contrast, our body mass estimates for the Mozambican *Endothiodon* specimens based on SC dimensions ranged from 116–182 kg (see above). Although they are in the same order of magnitude, the body mass estimates of SAM-PK-K11271 are more than triple our estimates for the Mozambican specimens. This difference may be due to the larger size of SAM-PK-K11271 when compared to the Mozambican *Endothiodon* specimens. Indeed, the measurement of the basioccipital condyle width (~6 cm) and of the width between the basal tubera (~9.5 cm) for SAM-PK-K11271 (Roger Smith personal communication) reveals that this specimen is significantly larger than the Mozambican specimens here described (~4 cm basioccipital condyle width and ~6 cm basal tubera width).

In the Late Permian of Mozambique, *Endothiodon* is among the most abundant and largest fossil taxa discovered to date. Thus, *Endothiodon* was likely a large-bodied herbivorous element of the late Permian terrestrial fauna occupying an ecological role similar to grazing mammals in African savannas today.

## Conclusions

Our study gives important new insights into dicynodont bony labyrinth anatomy. These findings raise new interesting questions about the biomechanics and functional morphology of the SCs. As CT-scanning technology becomes a widespread resource for paleontological research, more of the anatomy that was previously unclear due to classical preparation techniques is now becoming available. Thus, the morphology and variation of the non-mammalian synapsid inner ear is expected to shed light into the ecomorphology and systematic utility of this organ. The main conclusions of the present contribution are:

1. *Endothiodon* has low intraspecific variability of various biophysically relevant morphometric features, namely lumen diameter and angle between the SCs.
2. *Niassodon* and *Endothiodon* have highly eccentric semicircular canals when compared to a wide range of extant synapsids.
3. Within Endothiodontia, *Endothiodon* has unique vertically oriented SCs and an ellipsoidal horizontal SC.
4. *Endothiodon* was probably capable of performing fast head movements, probably during foraging and food processing.
5. Body mass estimates using inner ear morphology in dicynodonts may be more widely applicable than other proxies because: (i) it offers a proxy that can be sampled in a broader array of dicynodont taxa when compared to other methods (as dicynodont postcranial material is rarer); and (ii) there is a wide range of available information on the inner ear of mammalian taxa; (iii) the equations for estimating body mass based on SC dimension are very robust for modern mammals.
6. Given its abundance in the Mozambican Karoo deposits, *Endothiodon* must have been one of the large (>100 kg) dominant herbivorous members of the late Permian fauna from Mozambique.

## Supporting information

S1 Text History of Karoo vertebrate collection in Mozambique and AMNH6156 ascription to *Endothiodon*. The various important moments of Mozambican Karoo vertebrate paleontology are outlined starting 1949 with Domingos da Rocha to the 1980’s by Brigadas de Cartografia Geológica da Bacia carbonífera de Metangula.
(DOCX)

S1 Table Linear measurements and respective summary statistics calculations. (XLSX)

S2 Table Angular measurements and respective summary statistics calculations. (XLSX)

S3 Table Extant mammal and *Endothiodon* SC minor and major axis measurements, eccentricity calculations, body mass estimates and linear regression calculations and respective statistics. (XLSX)

S1 Fig Stereolitograph file of the MTA/ACL001 inner ear. (STL)

S2 Fig Stereolitograph file of the MTA/ACL002 inner ear. (STL)

S3 Fig Stereolitograph file of the MTA/ACL003 inner ear. (STL)
