# Supplementary material for: Endothiodon cf. bathystoma (Synapsida: Dicynodontia) bony labyrinth anatomy, variation and body mass estimates
Source: PLoS One. 2018 Mar 14;13(3):e0189883. doi: 10.1371/journal.pone.0189883 (PMC5851538; doi:10.1371/journal.pone.0189883)
Supplement: S1 Text — The various important moments of Mozambican Karoo vertebrate paleontology are outlined starting 1949 with Domingos da Rocha to the 1980’s by Brigadas de Cartografia Geológica da Bacia carbonífera de Metangula. (DOCX) [file pone.0189883.s001.docx]

SUPPLEMENTAL DATA

HISTORY OF KAROO VERTEBRATE COLLECTION IN MOZAMBIQUE

A succession of sedimentary units ascribed to the Karoo system largely outcrop in Mozambique, namely at the Tete and Niassa provinces. They soon arose much interest, mainly because they comprise several productive coal seams. The first reports of coal in the Niassa province go as far back as at the time of David and Charles Livingstone explorations [1]. They mention some coal fragments near the confluence between the Tiambila River and the Rovuma River [1]. However, later Stockley [2] noticed that those coal fragments came from the seams at the Mhukuru Basin in Tanzania. Nevertheless, Thompson [3] while describing for the first time the stratigraphy of the Mhukuru Basin noticed that there were coal remains in the Lunho River valley, Niassa.

The perhaps more complete succession has been recognized near the eastern shore of the Niassa Lake and further north-eastwards until Tanzania. The Karoo series there was described by some authors [4] as *Série de Lunho* comprising eleven main lithological units: conglomerates, sandstones and clayey beds more or less converted into slates, as well as coal beds.

Some levels (division 7) yielded plant remains (leaves, cuticles, fructifications) representative of the *Glossopteris* flora, so characteristic of the former Gondwana landmasses, and mainly the corresponding lower levels, Ecca/ Carboniferous and Permian in age [5].

Higher, upper Permian levels also proved to be fossil-bearing. Field work carried on by the then *Serviços de Geologia e Minas de Moçambique* under the direction of Fernando Mouta resulted into the discovery of bones in some clayey, grey or reddish beds with limestone concretions (division 10 of the same Lunho Series).

As far as we can ascertain, earlier collecting of fossil vertebrates was carried on three times.

**1949**

The first bone remnants were recovered by Mr. Domingos Rocha in the area between the Lunho [written *Lugno* in papers in English] river and its tributary Lundo just upstream of their confluence, i.e. about 30 km NNE from Maniamba, a village ca. 30 km NNE from Metangula, on the shore of the Lake Niassa [written *Nyasa* in papers in English]. Specimens were sent to Bernard Price Institute, Johannesburg, South Africa. Dr. Sidney Henry Haughton, paleontologist at the South African Museum, examined the concerned fossils and produced but a brief, unpublished report on them. Scant information became available, namely the occurrence of some Endothiodont and an Anomodont, an association which would suggest a Beaufort, Late Permian age.

**October 1956 and later comments on it**

As Haughton asked the Mozambique Geological Survey for additional collecting, Mr. Rocha searched again the same area. About 800 further bone specimens were recovered and sent for study soon afterwards. This collection was also examined by Haughton and a few references on its contents were published on a broad scope paper on Karoo Age reptiles [6].

Nevertheless, no study specifically on this material seems to have appeared, except for a short, unpublished report written by S. Haughton in 1957 with attached drawings as stated but have not been available to us. Here he states:

*« Most of the material, in so far as it is identifiable, consists of remains of one or more species of a large Endothiodont ».* …

*« Other fragments show the presence of animals of smaller size; but there is insufficient evidence to show whether these are lower jaws of a smaller species or are juvenile ».* …

*« The Anomodont material which forms by far the greater part of the collection is grey in colour and to much of it pieces of fine-grained hard calcareous nodular matrix are still attached. The collection, however, also contains a number of bone fragments which are almost free of matrix, are a pale reddish-brown in colour with a glazed surface, and which obviously come from a different type of reptile and a different type of deposit.* … *This well-preserved material, together with a fragment of a right dentary carrying a broken canine tooth and a smaller replacing canine immediately medial to it (No. 364) indicates the presence of a medium-sized Therapsid reptile which may be an advanced Gorgonopsian. The probability is that such an animal come from a higher stratigraphic horizon than the large Endothiodonts in the collection ».*

The same report had a few handwritten comments by J. W. Kitching, dated 19/6/91:

*« The specimens were unpacked, sorted and classified by J. W. Kitching during May and the first half of June 1991. The underlined numbers indicate that the specimens were missing when the wooden packing cases were unpacked ».*

Summing up, according to Haughton most of the specimens are from large endothiodontid, and a few ones from a middle-sized therapsid, the latter probably from a higher stratigraphic horizon.

**Late 1961 and later developments**

The previous failures to ensure a more complete study on these matters prompted Professor Carlos Teixeira to ask for new collecting that was carried on, as before, by Mr. Rocha. Material is quite numerous and more or less well preserved. It was sent to Lisbon, where Miguel Telles Antunes was charged to ensure its study, for which he got support from geologist Artur Figueiredo Nunes as far as stratigraphy is concerned.

The paleontological study was carried on in Paris at the Institut de Paléontologie of the Muséum national d’Histoire naturelle. The results were presented (June 4-9th 1973) in the Centre National de la Recherche Scientifique Colloquium held at Paris (Muséum), and published later [11]. It was indeed the first paper specifically on vertebrates from the Karoo units in Mozambique.

We concluded then that the fauna from the division 10 of the Lunho Series comprises:

- a *Endothiodon* cf. *bathystoma* Owen 1876;

- another anomodont not represented among the material studied (according to Haughton after some quite vague bibliographic references, if these can be corroborated);

- a rare, large-sized Gorgonopsian of an indeterminate genus and species.

As a whole, the fauna indicates as age the *Endothiodon* zone of the Beaufort Series, Upper Permian, an opinion that had been presented before but without clear evidence. That would be the age of the division 10 of the Lunho Series.

Meanwhile, Paleobotanical studies on the Karoo stratigraphic units from Mozambique show that associations comprise several *Glossopteris* species, *Gangamopteris, Paracalamites, Umbellaphyllites* and other remnants. However these associations concern the lower, ‘Productive series’ in the Tete region, correlated since long ago to the Middle Ecca flora of South Africa, Permian in age [7]. As far as we can ascertain, no paleobotanical data concerning the later (Beaufort Stage) levels with vertebrates are known.

As a concluding remark, the still far from completely known reptilian assemblage seems to more or less correspond to the *Tropidostroma* Assemblage Zone or a little later to the *Cistecephalus* A.Z. In comparison with South Africa east of 24^o^ E, the fossil-bearing deposits under study may be correlated to Middleton Formation, middle Beaufort Series, Upper Permian in age.

**1977 to 1980 and the “Brigadas de Cartografia Geológica da Bacia carbonífera de Metangula”**

The III FRELIMO Congress instructed the “Direcção Nacional de Geologia e Defesa do Subsolo” to initiate the studies at Metangula Graben [8]. Led by the geologists J. Verniers (1977-78) and R. V. Paulis (1979-80), the “Brigadas de Cartografia Geológica da Bacia carbonífera de Metangula” began their works at the Metangula Graben with the purpose of evaluating the economic potential of the coals present in the lower section of the basin and cartograph at a 1:40.000 the geology of the region [9]. They found that the fossiliferous formations were K6 and K5. K6 is further subdivided into K6b with “red or black bones” preserved in a red siltstone and, there are “black bones” preserved in a grey siltstone-sandstone at the top of K6a [10]. Jourdan and Verniers [9] indicated that the fossils studied by Antunes [10] came from the K5 Formation.

AMNH6156 AS AN *ENDOTHIODON*


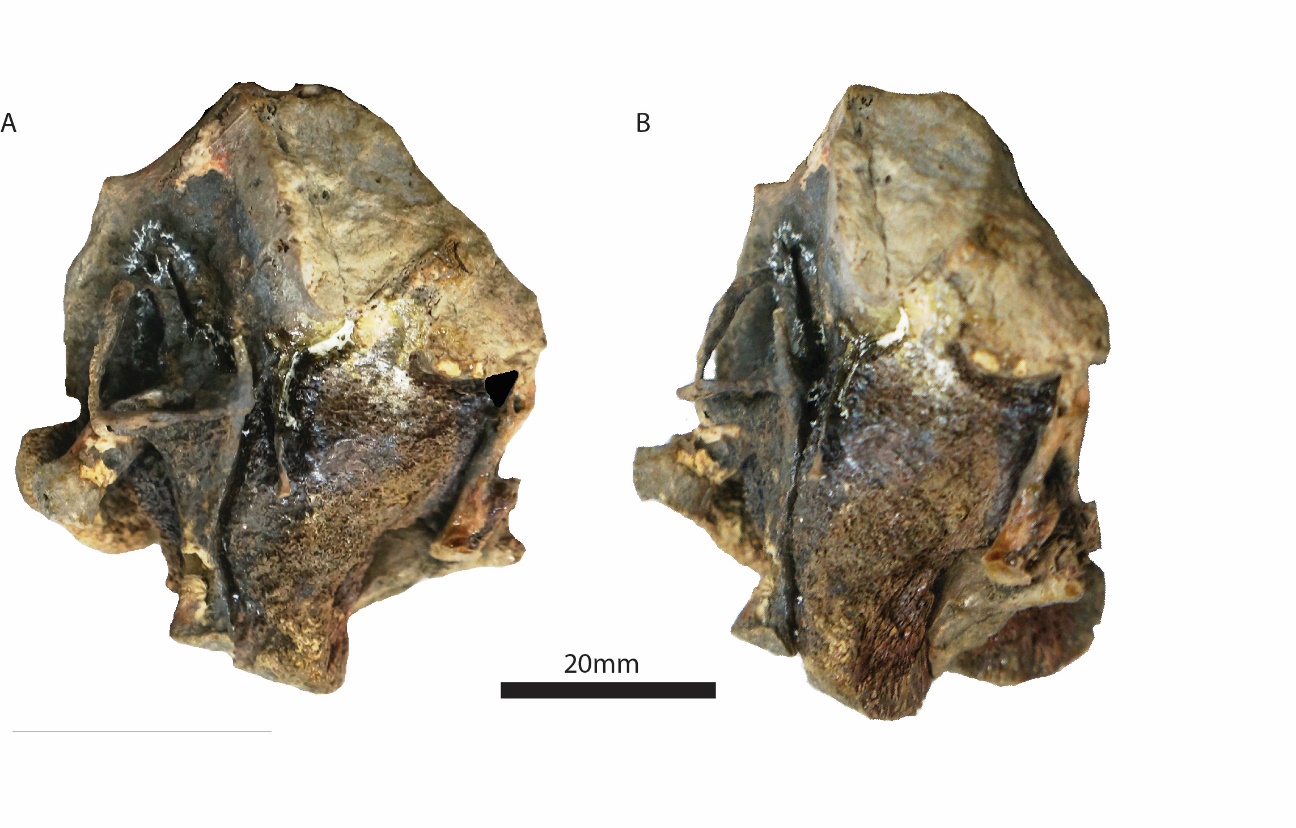


Supplemental Figure 1 – AMNH6156 natural endocast in **A**, lateral view and **B**, anterior view.

The AMNH6156 osseous labyrinth natural endocast (Supplemental Figure 1, [11, 12]) is strikingly similar to those of the *Endothiodon* specimens here described. The vertically elongated anterior and posterior SCs, the triangular base of the crus communis, the elliptical lateral SC or the long subcylindrical vestibule are some of the most directly comparable features demonstrating the similarity between AMNH6156 and the specimens here described. *Kawingasaurus* (GPIT/RE/9272) differs from *Endothiodon* because it possesses an inflated vestibule and the size of the SCs is much smaller [13]. *Pristerodon* (MBR985) osseous labyrinth is significantly smaller than that of *Endothiodon*, and has well-developed ampullae [14]. Nevertheless, the anterior and posterior SCs are also relatively eccentric [14]. *Brachyprosopus* although having a subtriangular crus communis, has well-developed ampullae and relatively subcircular SCs [15]. Other published dicynodont descriptions have been obtained via serial grinding (e.g., [16-18]), thus rendering questionable measurements because a description of the methodology is not detailed (e.g., interslice distance).

Broom’s biozonation scheme [19] correlates to the more restricted edition of the same assemblage zone today [20, 21]. AMNH6156 is thus from the penecontemporaneous biozone of the Mozambican *Endothiodon* specimens [14, 21]. Therefore, we tentatively ascribe AMNH6156 to *Endothiodon.* Initially, Broom [12] ascribed the specimen to *Dicynodon* ?*bolorhinus*, but it was later considered as undiagnosable to genus level [11]. The holotype of *Dicynodon bolorhinus* (AMNH FARB 5505) is a weathered partial snout [22] which prevents direct comparison with AMNH6156. In a recent revision of the genus *Dicynodon* [22], the species *Dicynodon* ?*bolorhinus* was considered as a junior subjective synonym of *Oudenodon bainii*. *Oudenodon* is contemporaneous to *Endothiodon*, however *Oudenodon* osseous labyrinth is currently unknown. Therefore the current ascription of AMNH6156 remains tentative until new information on the osseous labyrinth of *Oudenodon* is provided.

LITERATURE CITED

1. Livingstone D, Livingstone C. Explorations of the Zambezi (Narrative of an Expedition to the Zambezi and its tributaries. Gerald Duckworth & Co., London.

2. Stockley GM. New coal discoveries in Tanganyka. Mineralogical Mag. 1865; 76: 329-338.

3. Thompson J. Notes on the Basin of the River Rovuma, East Africa. Proc R Geogr Soc; 1882.

4. Borges A, Nunes AF, Freitas F. Contribution to the data on the Karroo of portuguese Lake Nyasa. Congr. Géol. Intem., comptes rendus 19c session, Alger, 1952. Ass Serv géol Afr. 1954; 21: 83-91.

5. Teixeira C, Gonçalves F. Contribuição para o conhecimento geológico do Karroo da África Portuguesa. IV—Descrição de alguns restos de vegetais do Karroo das regiões de Niassa e de Mossurize (Moçambique). Garcia Orta. 1959; 7: 785–789.

6. Haughton S. Note on the distribution of fossil reptilia of Karroo age. Palaeont Afr*.* 1963; 8: 1–11.

7. Paulis RV, Jourdan PP, Frasca Spada L. Relatório da Brigada de cartografia carbonífera de Metangula 1978-79. Relatório Inédito da Direcção de Geologia e Minas de Moçambique. 1980; 1122: 1–23.

8. Jourdan PP, Verniers J. Relatório final do Karroo da mancha de Metangula (Maniamba). Relatório da Direcçao Nacional de Geologia do Moçambique (Maputo)(= Professional papers of the Geological Survey of Mozambique), 81 p; 1983.

9. Verniers J, Jourdan PP, Paulis RV, Frasca-Spada L, De Bock FR. The Karroo Graben of Metangula Northern Mozambique. J Afr Earth Sci. 1989; 9: 137–158. doi:10.1016/0899-5362(89)90016-X

10. Antunes MT. Sur quelques reptiles du Karroo de Maniamba, Mocambique. Colloq. Int Cent Nat Rech Sci. 1975; 218: 371–378.

11. Cox CB. A natural cast of the inner ear of a dicynodont. Am Mus Novit. 1962; 2116:1–6.

12. Broom R. On the structure of the internal ear and the relations of the basicranial nerves in *Dicynodon*, and on the homology of the mammalian auditory ossicles. Proc Zool Soc Lond. 1912; 82: 419–425. doi: 10.1111/j.1469-7998.1912.tb07027.x

13. Castanhinha R, Araújo R, Júnior LC, Angielczyk KD, Martins GG, Martins RMS, Chaouiya C, Beckmann F, Wilde F. Bringing dicynodonts back to life: paleobiology and anatomy of a new emydopoid genus from the Upper Permian of Mozambique. PLoS One. 2013; 8: e80974

14. Laaß M. Bone‐conduction hearing and seismic sensitivity of the Late Permian anomodont *Kawingasaurus* *fossilis*. J Morphol. 2015; 276: 121–143. doi: 10.1002/jmor.20325

15. Angielczyk KD, Rubidge BS, Day MO, Lin F. A reevaluation of *Brachyprosopus broomi* and *Chelydontops altidentalis*, dicynodonts (Therapsida, Anomodontia) from the middle Permian *Tapinocephalus* Assemblage Zone of the Karoo Basin, South Africa. J Vertebr Paleontol*.* 2016; 36: e1078342. doi:10.1080/02724634.2016.1078342

16. Sollas IB, Sollas WJ. A study of the skull of a *Dicynodon* by means of serial sections. Phil Trans R Soc B. 1914; 204: 201–225.

17. Keyser AW. A preliminary study of the type area of the *Cistecephalus* zone of the Beaufort Series, and a revision of the anomodont family Cistecephalidae. S Afr Geol Surv Memoirs. 1973; 62: 1–71

18. Fourie H. A detailed description of the internal structure of the skull of *Emydops* (Therapsida: Dicynodontia). Palaeontol Afr. 1993; 30: 103–111

19. Broom R. On the Permian and Triassic faunas of South Africa. Geol Mag. 1906; 5: 29–30. doi: https://doi.org/10.1017/S001675680012271X

20. Rubidge BS. Biostratigraphy of the Beaufort Group (Karoo Supergroup). Biostratigraphic Ser S Afr Comm Stratigraphy Rep. 1995; 1: 3–7.

21. Rubidge BS, Erwin DH, Ramezani J, Bowring SA, de Klerk WJ. High-precision temporal calibration of Late Permian vertebrate biostratigraphy: U-Pb zircon constraints from the Karoo Supergroup, South Africa. Geology. 2013; 41: 363–366. doi: 10.1130/G33622.1

22. Kammerer, CF, Angielczyk KD, Fröbisch J. A comprehensive taxonomic revision of *Dicynodon* (Therapsida, Anomodontia) and its implications for dicynodont phylogeny, biogeography, and biostratigraphy. J Vertebr Paleontol. 2011; 31: 1–158. doi: http://dx.doi.org/10.1080/02724634.2011.627074
